# An Indian Medical Journal

**Published:** 1860-10

**Authors:** 


*An Indian Medical Journal* has been established in Oordoo, E. I., entitled the *Akbare Tubabut.* It is intended as a medium of communication between native doctors in government employ and native Hakims, for the improvement of medical and surgical knowledge, and the greater alleviation of the many diseases to which the millions of inhabitants of this country are subject. B.

